# The grain protein yield of barley under future drought is modified by the joint action of elevated CO_2_ and temperature

**DOI:** 10.1093/jxb/eraf531

**Published:** 2025-12-02

**Authors:** Ander Yoldi-Achalandabaso, Jon Miranda-Apodaca, Ismael Gutiérrez-Fernández, Marlon de la Peña, Usue Pérez-López, Alberto Muñóz-Rueda

**Affiliations:** Fisioklima-AgroSosT Group, Department of Plant Biology and Ecology, Faculty of Science and Technology, University of the Basque Country (UPV/EHU), Leioa, Spain; Fisioklima-AgroSosT Group, Department of Plant Biology and Ecology, Faculty of Science and Technology, University of the Basque Country (UPV/EHU), Leioa, Spain; Fisioklima-AgroSosT Group, Department of Plant Biology and Ecology, Faculty of Science and Technology, University of the Basque Country (UPV/EHU), Leioa, Spain; Department of Abiotic Stress, Institute of Natural Resources and Agrobiology of Salamanca (IRNASA), Spanish National Research Council (CSIC), Salamanca, Spain; Sugarcane Research Center of Colombia, CENICAÑA, Colombia; Fisioklima-AgroSosT Group, Department of Plant Biology and Ecology, Faculty of Science and Technology, University of the Basque Country (UPV/EHU), Leioa, Spain; Fisioklima-AgroSosT Group, Department of Plant Biology and Ecology, Faculty of Science and Technology, University of the Basque Country (UPV/EHU), Leioa, Spain; INRAE-Versailles, France

**Keywords:** C metabolism, drought, elevated [CO_2_], elevated temperature, grain protein, N metabolism, N status, N uptake, photorespiration, sink strength

## Abstract

Nitrogen is the pivotal macronutrient for grain protein synthesis, which is important in human nutrition and the establishment of derived products. However, nitrogen metabolism in plants is likely to be susceptible to abiotic factors such as those derived from climate change: drought, elevated [CO_2_], and temperature. How the triple interaction of these factors will affect nitrogen metabolism and grain quality of cereals is unknown. This study aimed to determine the response of nitrogen metabolism in barley—one of the temperate cereals most tolerant to abiotic stresses—to the triple interaction during its whole life span. Our results pointed out a growth stage-dependent response on final nitrogen status. At the vegetative stage, the nitrogen assimilation capacity was boosted and matched with the biomass gain without altering the nitrogen status. However, at the anthesis and maturity stages, the nitrogen status of plants and grains was reduced. A non-overlapping effect of biomass dilution and lower mass flow is highlighted, while lower photorespiration activity cannot be completely ruled out. The elevated [CO_2_] is the main driver regulating nitrogen metabolism at the physiological level under future drought conditions, whereas elevated temperature hampers grain formation.

## Teaser

Under the future drought scenario, elevated CO_2_ leads to an imbalance in the C/N status, affecting barley N metabolism as phenology progresses, while elevated temperature hinders grain set.

## Introduction

Nitrogen (N) is the pivotal macronutrient for cereal yield and grain quality establishment as it is the main component of amino acids, the building blocks of proteins in plants (Zayed *et al*., 2023). Plants take up N predominantly as nitrate, by specific transporters, through their root system. N is subsequently reduced to ammonium either in the roots or in the leaves. Nevertheless, this reduction is mainly accomplished in leaves by the action of nitrate reductase (NR) and nitrite reductase (NiR). This ammonium is generally assimilated into amino acids by the concomitant glutamine synthetase/glutamate synthase cycle (GS/GOGAT) (Balotf *et al*., 2016). Alternatively, plants have been observed to assimilate ammonium by glutamate dehydrogenase activity (NADH-GDH) when GS/GOGAT is inhibited under stress conditions (Robredo *et al*., 2011). However, the main role of this enzyme has been identified as deaminating glutamate (NAD-GDH) into ammonium and 2-oxoglutarate (2-OG) (Vega-Mas *et al*., 2024).

Overall, these processes require energy, reducing power, and carbon (C) skeletons derived from photosynthesis, respiration, and photorespiration, which are closely linked to N metabolism (Masclaux-Daubresse *et al*., 2010). As with other physiological processes, N metabolism in plants is susceptible to being affected by abiotic factors such as those derived from climate change. In this respect, the IPCC foresees more complex abiotic scenarios for the second half of the 21st century: air [CO_2_] values of ∼700 ppm and an average increase in earth surface temperature of 3 °C, together with an increase in the duration and severity of drought periods in the Mediterranean agro-environment (IPCC, 2021). Such conditions are predicted to have a detrimental effect on cereal production and quality, and to pose a threat to global food security (Araus, 2004). In this regard, barley is proposed as a target temperate cereal to deal with climate change constraints (Kebede *et al*., 2019).

These environmental factors influence N metabolism differently depending on the plant species, the developmental stage, the plant organ, and the intensity and duration of exposure to the stress (Ivanov and Kosobryukhov, 2018; Tausz-Posch *et al*., 2020). In this regard, on the one hand drought and elevated temperature (ET; above the optimum temperature) constrain the N status of the plant. Drought decreases N uptake through the reduction of water flow caused by stomatal closure, which also affects CO_2_ uptake, reducing the net photosynthetic rate. Consequently, due to the lower availability of N, and lower energy and C skeletons supplied, the reduction and assimilation of N decreases, leading to a decline in the synthesis of free amino acids (FAAs) and proteins in cereals (Fresneau *et al*., 2007; Ashoub *et al*., 2015). At ET, water and N flow are more likely to increase due to higher transpiration (Sadok *et al*., 2021). At the same time, the ET alters the thermostability and activation state of the enzymes involved in not only N metabolism, but also C metabolism (e.g. Rubisco). Consequently, photosynthesis and subsequent N reduction and assimilation are affected (Yuan *et al*., 2017). In the long term, lower photosynthetic rates can constrain spikelet viability and/or grain filling during grain formation, which can alter grain N status and quality (Barnabás *et al*., 2008; Högy *et al*., 2013).

On the other hand, the effect of elevated air CO_2_ concentration (ECO_2_) on N metabolism has been widely investigated. However, the implications are still uncertain and highly variable depending on the species, cultivar, developmental stage, and organ (Feng *et al*., 2015). In general, ECO_2_ decreases the leaf N concentration in C_3_ species (Wang *et al*., 2012; Perkowski *et al*., 2025), but the mechanisms behind this reduction remain unclear. In this regard, three main hypotheses have been proposed (Tausz-Posch *et al*., 2020). First, it has been hypothesized that when plants cannot match N requirements with growth, increased biomass results in a dilution effect, giving as a result a lower [N] (Taub and Wang, 2008). Secondly, the mass flow hypothesis suggests that the main cause of the lower N concentration is the reduced N uptake by the roots, triggered by stomatal closure and therefore lower mass flow (Van Vuuren *et al*., 1997). Thirdly, the last hypothesis refers to the physiological readjustment of plant N metabolism under ECO_2_, which would be highly linked to development of putative photosynthetic acclimation (PAC) (Cui *et al*., 2023). Such acclimation results in a shift in N investment from the photosynthetic enzymes to other functions, such as defence and structure (Bassiouni *et al*., 2025), and to lower photorespiratory levels (Bloom, 2015: Busch *et al*., 2018; Zhao *et al*, 2021). Nevertheless, this hypothesis is the most controversial one and there is no consensus around it (Andrews *et al*., 2019). In any case, grain protein concentration appears to decrease under ECO_2_ conditions (Myers *et al*., 2014). Depending on the final use of the grain, this fact could jeopardize the nutritional quality of the grains for human nutrition and animal feeding (Tausz-Posch *et al*., 2020), but increase the quality for the brewery industry (Erbs *et al*., 2010).

Moreover, stresses in nature tend to occur together, as in the case of combined drought and ET conditions. Under this scenario, the photosynthetic activity is more likely to be affected than under single conditions (Wang *et al*., 2010). Consequently, the N status of plants is more impaired than under single effects (Liu *et al*., 2017). Overall, the combined action of these factors results in lower grain quality properties (Högy *et al*., 2013). In contrast, research on the interaction between factors such as drought or ET and ECO_2_ is scarce. In the case of the interaction between drought and ECO_2_, ameliorative effects have been recorded in barley with regard to N assimilation capacity and N status (Robredo *et al*., 2011), as well as grain formation (Schmid *et al*., 2016), compared with the effect of drought alone. Regarding the combined ET×ECO_2_ conditions, more heterogeneous results have been obtained due to the uncertainty of the response of plant N metabolism to ECO_2_, where the development or absence of PAC seems to play a pivotal role. Specifically, either a better response (Dias de Oliveira *et al*., 2015b) or a worse response (Jauregui *et al*., 2015) has been observed compared with single stresses. At the grain protein level, the same protein concentration as under current air CO_2_ concentration conditions (ACO_2_) has been observed in >100 barley cultivars. However, lower harvestable protein has been observed owing to the negative effects of ET on grain formation, although the response was cultivar dependent (Ingvordsen *et al*., 2016).

To the best of our knowledge, to date no study has addressed how the complex triple interaction of the main climate change drivers (drought×ET×ECO_2_) affects the N metabolism of cereals (Qaderi *et al*., 2025). Only Zinta *et al*. (2018) have analysed this, albeit by considering only amino acid and protein content in Arabidopsis. Furthermore, most studies analysing the triple interaction effect on cereal physiology focus primarily on the vegetative stage rather than on the reproductive stage. In other words, few studies have analysed the physiological response of cereals across key growth stages up to physiological maturity, which is a critical phase (Yoldi-Achalandabaso *et al*., 2025). Given that cereal production is an important economic activity with priceless nutritional value in agro-environments such as the Mediterranean (PRIMA, 2022), it is critical to understand how N metabolism of cereals will respond to this likely scenario at different key growth stages in order to decipher potential adaptive mechanisms and crop management strategies that can ensure food security. In view of the shortcomings regarding the impact of the triple interaction, the present study aimed to analyse how the N status of barley responds to the future triple interaction of drought, ECO_2_, and ET conditions at vegetative, anthesis, and maturity stages. To gain a comprehensive understanding, the study examined the uptake, reduction, and assimilation capacity of leaves and roots, as well as leaf photosynthesis, dark respiration, and photorespiration activity, and their influence on the N status of the plants. We hypothesize that drought-related constraints on N uptake, reduction, assimilation, and status in barley will be alleviated by the combined effect of ECO_2_ and ET at vegetative and anthesis stages, due to the fertilizing effect of ECO_2_ on photosynthesis. Regarding grain protein concentration, this will decrease compared with current conditions due to a dilution effect caused by ECO_2_, which improves grain quality for the brewing industry. However, ET will hinder grain formation.

## Materials and methods

### Plant material and growing conditions

A modern malting barley cultivar (*Hordeum vulgare* cv. Henley) with demonstrated drought tolerance (Yoldi-Achalandabaso *et al*., 2024) and high productivity in arid and semi-arid climatic conditions (GENVCE; https://genvce.org/wp-content/uploads/2019/12/Henley.pdf) was used in this experiment. Growing conditions are explained in Yoldi-Achalandabaso *et al*. (2024). Briefly, plants were grown in 3.1 litre pots with a 3:1 mixture of perlite/vermiculite and a sowing density of 350 plants m^−2^ in an environment-controlled growth chamber (Conviron PGR15; Conviron, Manitoba, Canada). The photosynthetic photon flux density was set to 400 µmol m^−2^ s^−1^ (14 h/10 h day/night), provided by a combination of warm-white fluorescent lamps and incandescent light bulbs (Sylvania F48T12SHO/VHO, Sylvania, USA). The humidity was maintained at 70%/80% day/night. To minimize the effects of intra-chamber environmental gradients, plants were randomly repositioned within the chamber each week.

#### CO_2_ and temperature conditions

Two atmospheric concentrations were set up: current air [CO_2_] at the time this study was conducted (CA), 400 ppm, and elevated air [CO_2_] (CE), 700 ppm Likewise, two temperature regimens were used during day/night: ambient temperature (TA), 23/17 °C, and elevated temperature (TE), 26/20 °C. In total, barley plants were grown under four different environmental conditions: current CO_2_ and temperature (CATA), current CO_2_ and elevated temperature (CATE), elevated CO_2_ and current temperature (CETA), and elevated CO_2_ and temperature conditions (CETE). The CO_2_ and temperature conditions were set up from sowing till maturity.

#### Plant watering and drought-imposed conditions

Twice per week plants were irrigated with Hoagland’s solution (Arnon and Hoagland, 1940) alternating with deionized water. Twenty-one d after sowing, coinciding with the onset of tillering [growth stage (GS) 21], all pots were adjusted to field capacity with deionized water. Half of them were deprived of water for 9 d (VD), whereas the other half was maintained at field capacity (VC) until GS30. From this point till the next drought period, plants were irrigated with Hoagland’s solution and deionized water.

When half of the plants of each pot reached anthesis (GS51), the anthesis drought period began. Based on Sreenivasulu and Schnurbusch (2012), this growth stage in barley was associated with awn tipping. As in the vegetative stage, all pots were watered to field capacity before drought imposition. During 9 d, half of the plants that were maintained at optimum watering conditions during the vegetative stage were watered with 50% of the daily plant evapotranspiration rate (AD). The other half was maintained at field capacity (AC). Subsequently, irrigation was gradually decreased to induce natural senescence until AC and AD plants reached physiological maturity.

### Determination of N and C assimilation

#### Determination of plant [N] and N acquisition

The organ and plant N concentration was measured using an elemental analyser (FlashEA 1112; ThermoFinnigan, Germany), measuring leaf N concentration ([leaf N]), root N concentration ([root N]), and plant N concentration ([plant N]). In addition, organ N content was obtained by multiplying the [N] by the organ biomass: leaf N content (LNC), root N content (RNC), and plant N content (PNC). The material employed for determination of this parameter came from the total organ dry weight of each biological replicate for each treatment. For the specific case of grains, according to Mariotti *et al*. (2008), the protein concentration ([grain protein]) was estimated from the N concentration by applying the Jones factor of 5.45 for barley. Furthermore, the grain protein content (TGP) and the yield grain protein content (YGP) were calculated by multiplying the grain protein concentration by the individual grain weight (IGW) and plant yield, respectively.

N availability was analysed by determination of the N uptake rate (NUR) and N translocation rate (NTR) (Franklin and Zwiazek, 2004). NUR and NTR were calculated by:


$$\mathrm{NUR}\;\left(\frac{\mathrm{mgN}}{g\;\mathrm{rootDW}\;\times d}\right)=\frac{(N_{\mathrm{tot}2}-N_{\mathrm{tot}1})\times (\mathrm{Ln}\left(\frac{W_{r2}}{W_{r1}}\right)}{\left(T_{2}-T_{1})\times (W_{r2}-W_{r1}\right)}$$

$$\mathrm{NTR}\;\left(\frac{\mathrm{mgN}}{g\;\mathrm{rootDW}\;\times d}\right)=\frac{(N_{\mathrm{aer}2}-N_{\mathrm{aer}1})\times (\mathrm{Ln}\left(\frac{W_{r2}}{W_{r1}}\right)}{\left(T_{2}-T_{1})\times (W_{r2}-W_{r1}\right)}$$

where N_tot1_ refers to the plant N content at the beginning of the drought treatment (T_1_), N_tot2_ to the plant N content at the end of the drought treatment (T_2_), T is the time in days, and W_r_ refers to the root dry weight at the beginning (1) and end (2) of the drought treatment. Likewise, N_aer1_ and N_aer2_ refer to the aerial N content at the beginning and end of the drought periods, respectively.

#### Quantification of enzyme activities related to N metabolism

For leaf and root NR extraction and determination of maximum and actual activities (NRmax and NRact), the procedure described by Robredo *et al*. (2011) was followed, updated by adding 5 mM 5′-AMP to the extraction buffer of NRmax to fully activate it (Abd-el Baki *et al*., 2000). Leaf and root GS, NADH-GDH, and NAD-GDH enzyme extraction and quantification were carried out following Robredo *et al*. (2011). GOGAT extraction was carried out using the same extraction buffer as for the latter, while its determination was done following Coleto *et al*. (2017).

#### Quantification of nitrate, ammonium, free amino acids, and soluble proteins

The determination of nitrate (NO_3_^−^), FAAs ([FAAs]), ammonium (NH_4_^+^), and total soluble protein ([TSP]) was done spectrophotometrically at 210, 570, 635, and 590 nm, respectively. Briefly, NO_3_^−^ was extracted with 2% (w/v) sulfamic acid and determined following Cawse (1967). The FAAs and NH_4_^+^ were extracted with 2% (w/v) sulfosalicylic acid. The former was determined following Deepa *et al*. (2003), while the latter was quantified by the method of Yemm *et al*. (1955). The TSPs were measured according to Bradford (1976).

#### Quantification of traits related to C metabolism

The net CO_2_ assimilation rate (*A*_net_) was determined using the Li-Cor 6400 system. The youngest fully expanded leaf of the principal tiller was used for the analysis, recording the measurements between 2 h and 3 h after dawn. Cuvette conditions were specifically set up for each environmental condition, mimicking the temperature and [CO_2_] of the the growth conditions for all the treatments. The light photosynthetically active radiation (PAR) and humidity were maintained constant for all the treatments (400 PAR and 60% relative humidity). Data were saved at water and CO_2_ steady-state exchange, ∼10 min from the onset of the measurement. In addition, the night respiration rate (*R*_n_) was also determined before dawn under the same humidity and CO_2_ conditions, but the PAR was maintained at 0 μmol m^−2^ s^−1^ and the employed temperatures were the night temperatures of the experiment (17 °C for CATA and CETA, and 20 °C for CATE and CETE). Leakage from the cuvette was taken into account for both *A*_net_ and *R*_n_ calculation. Moreover, the percentage leaf senescence (senescent %) was obtained by subtracting the greenness percentage from 100%.

Lastly, two enzymes involved in the photorespiration pathway were measured in leaves: hydroxypyruvate reductase (HPR) and glycolate oxidase (GO). Enzyme extraction and determination were both carried out as detailed in Vega-Mas *et al*. (2024).

### Statistical analysis

The pot was considered as the biological experimental unit. The mean ±SE for each trait and treatment was obtained by using four independent biological replicates. Two pools of samples were analysed per pot as technical replicates. To evaluate the main effects of the studied factors on all dependent traits, a three-way ANOVA was carried out using the SPSS 24.0 software package (IBM Corp., USA). The homogeneity and normal distribution were checked by Levene’s and Kolmogorov–Smirnov tests, respectively. Tukey’s multiple-range test was used to compare between means. Principal component analysis (PCA) was performed on ClustVis, autoscaling the data (Metsalu and Vilo, 2015), and Metaboanalyst 6.0 was used for partial least squares discriminant analysis (PLS-DA) to identify key traits [variable importance in projection (VIP) >1 and *t*-test <0.05] that discriminate between plant groups with high and low protein content (Pang *et al*., 2024). For the latter, data were transformed using square root transformation, followed by Pareto scaling.

## Results and discussion

### The environmental condition drives the N dynamics of barley under drought and the response at CETE is growth stage specific

In the present study, we investigated the response of barley N metabolism to drought under future conditions, combining ET and ECO_2_ (CETE), research that has not been carried out on any cereal to date. To this end, we studied the physiological effect of drought on barley N metabolism at the vegetative (VD) and anthesis (AD) stages, taking the plants up to maturity to investigate its effect on grain quality. When plants sense the effect of drought on the soil through the roots, they primary trigger an abscisic acid (ABA)-driven signalling cascade (He *et al*., 2024) that mainly results in stomatal pore closure (Hsu *et al*., 2021). Indeed, stomatal closure is a general mechanism turned on by barley plants to cope with drought, regardless of the level of stress, as demonstrated in our previous work (Yoldi-Achalandabaso *et al*., 2024).

**Fig. 1.**
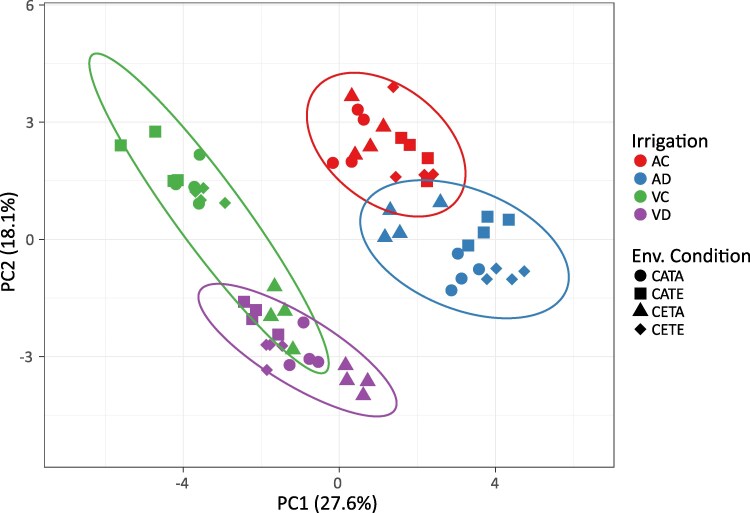
Principal component analysis (PCA) of the effect of the studied main factors on N metabolism variables. The main factors studied were leaf and root N, TSP and FAA concentration, plant N concentration, NUR, NTR, leaf and root NRmax, NRact, GS, GOGAT, NADH-GDH, and NAD-GDH activities, leaf HPR and GO activities, and leaf and root nitrate and ammonium concentration. The abbreviations for the variables are explained in the text. Anthesis control (AC) and anthesis drought (AD) relate to the anthesis growth stage, and vegetative control (VC) and vegetative drought (VD) relate to the vegetative growth stage, respectively. The environmental conditions were as follows: CATA, current CO_2_ level and temperature; CATE, current CO_2_ level and elevated temperature; CETA, elevated CO_2_ and current temperature; CETE, elevated CO_2_ and temperature.

**Fig. 2.**
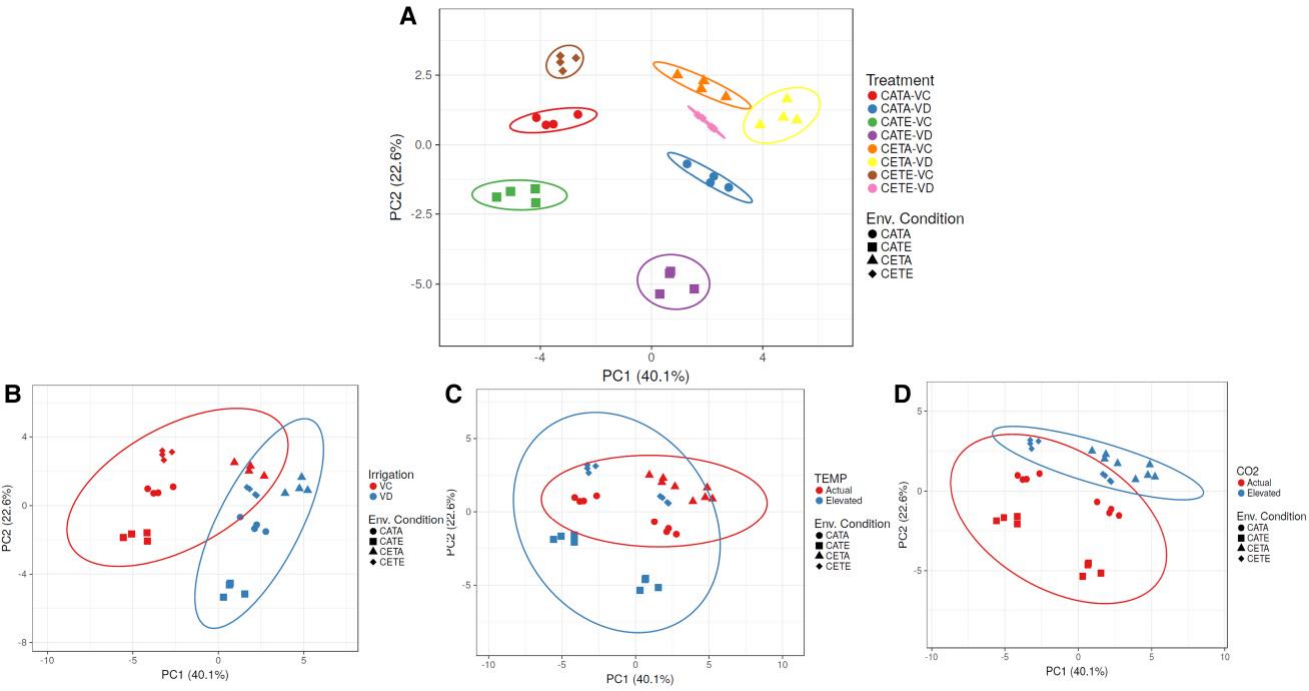
Principal component analysis (PCA) of the effect of the studied main factors on N metabolism variables. (A) The effects on leaf and root N, TSP and FAA concentration, plant N concentration, NUR, NTR, leaf and root NRmax, NRact, GS, GOGAT, NADH-GDH, and NAD-GDH activities, leaf HPR and GO activities, and leaf and root nitrate and ammonium concentration at the vegetative stage. VC, vegetative control; VD, vegetative drought. (B, C, and D) The PCA sorted by the irrigation, temperature, and air CO_2_ concentration levels. The abbreviations for the variables are explained in the text. The environmental conditions were as follows: CATA, current CO_2_ level and temperature; CATE, current CO_2_ level and elevated temperature; CETA, elevated CO_2_ and current temperature; CETE, elevated CO_2_ and temperature.

**Fig. 3.**
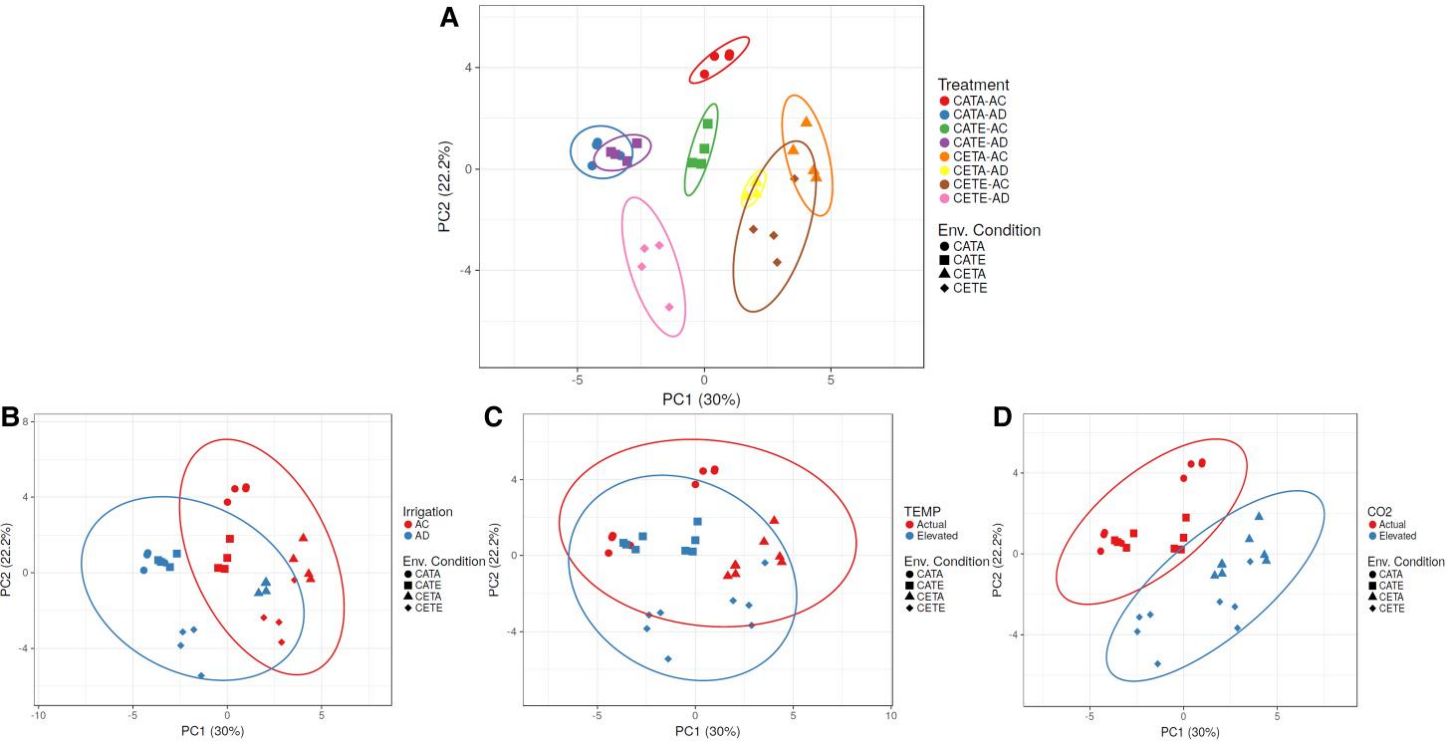
Principal component analysis (PCA) of the effect of the studied main factors on N metabolism variables. (A) The effects on leaf and root N, TSP and FAA concentration, plant N concentration, NUR, NTR, leaf and root NRmax, NRact, GS, GOGAT, NADH-GDH, and NAD-GDH activities, leaf HPR and GO activities, and leaf and root nitrate and ammonium concentration at the anthesis stage. AC, anthesis control; AD, anthesis drought. (B, C, and D) The PCA sorted by the irrigation, temperature, and air CO_2_ concentration levels. The abbeviations for the variables are explained in the text. The environmental conditions were as follows: CATA, current CO_2_ level and temperature; CATE, current CO_2_ level and elevated temperature; CETA, elevated CO_2_ and current temperature; CETE, elevated CO_2_ and temperature.

As we will explain later, irrespective of environmental conditions, the reductions in stomatal conductance resulted in reduced N uptake, reduced photosynthetic rates, and impaired N reduction and assimilation, the latter mainly driven by the down-regulated activity of leaf and root NR. This general response of barley is consistent with the literature (Robredo *et al*., 2011; Ashoub *et al*., 2015) and for other relevant cereals such as wheat (Fresneau *et al*., 2007). However, the effect of drought on barley N status and N metabolism varied over the development stages (Fig. 1). Interestingly, at the vegetative stage there was a clear separation between drought and irrigated treatments (Fig. 2A–D), while this separation disappeared at anthesis, relegating the importance of drought treatment in contrast to ECO_2_ and ET (Fig. 3A–D). In the case of CETE, the N status was growth stage specific and the causes behind this were varied, with ECO_2_ being the environmental factor that governed the physiological response of CETE, while the ET led the response of grain formation and the protein content. These statements will be expanded upon in the following description and discussion of the results.

### At the vegetative stage, the N assimilation under CETE is boosted, but not the N status

**Fig. 4.**
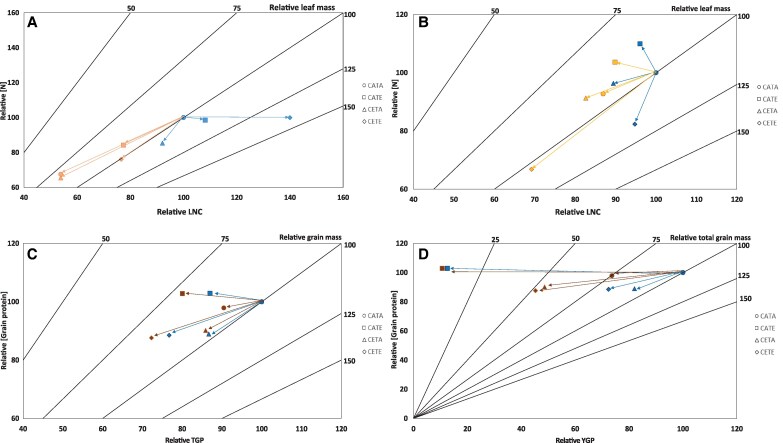
Graphical vector analysis (GVA) of the effect of drought, ET, and ECO_2_. Effect on the leaf N concentration [leaf N], leaf N content (LNC), and leaf biomass at the vegetative stage (A) and anthesis stage (B), on grain protein concentration ([grain protein]), grain protein content (TGP), and individual grain weight (IGW) at the maturity stage (C), and on yield grain protein content (YGP), [grain protein], and total grain mass (D). All the values are relative to CATA well-watered treatment (100,100). In blue, well-watered treatments during the different growth stages (VC, AC, and MC); in orange, VD treatments; in yellow, AD; and in brown, MD treatments. The environmental conditions are shown in the key.

**Fig. 5.**
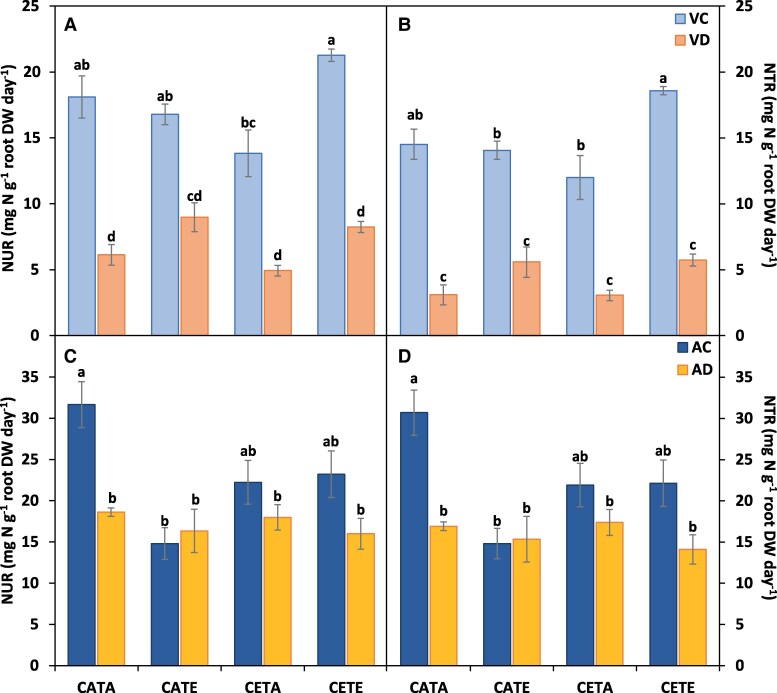
Nitrogen uptake rate (NUR) and nitrogen translocation rate (NTR) of barley plants. (A, B) The vegetative stage and (C, D) the anthesis stage. Barley plants were subjected to different environmental conditions (CATA, current CO_2_ and temperature; CATE, current CO_2_ and elevated temperature; CETA, elevated CO_2_ and current temperature; CETE, elevated CO_2_ and temperature) and water regimes (VC, vegetative control; VD, vegetative drought; AC, anthesis control; AD, anthesis drought). Values represent the mean ±SE of four biological replicates. Different letters indicate significant differences (*P*≤0.05) between water regime treatments and the environmental conditions. Values were analysed separately for the vegetative and anthesis stages.

At the vegetative stage, drought was identified as the main environmental factor (Fig. 2B–D). Overall, it split the treatments into two groups—except for CETA-VD—as denoted in the PCA (Fig. 2A). However, regardless of the environmental condition, it was classified as mild drought based on the null effect on the leaf relative water content and the slight reduction in the leaf water potential and stomatal conductance observed in a previous study (Yoldi-Achalandabaso *et al*., 2024). CETE conditions did not modify the barley N status compared with CATA when suffering a drought period at the vegetative stage (VD; Table 1). Specifically, CETE did not alter leaf and root N concentration or protein concentration, although the [leaf FAA] (Table 1) and LNC (Supplementary Table S1) were increased by 60% and 40%, respectively. This was due to the higher leaf biomass produced due to the increased photosynthetic rates (Table 2). The lack of differences in the above-mentioned parameters would indicate that CETE plants were able to match the N requirements with the biomass gain as observed in the graphical vector analysis (GVA; Fig. 4A). In agreement with our results, Sinclair *et al*. (2000) also observed the same [leaf N] levels for wheat during 3–4 growing seasons in a free-air CO2 enrichment (FACE) experiment—only studying the effect of ECO_2_– under irrigated and rainfed conditions. Although this is not the main overall response observed in the literature for the effect of ECO_2_ on leaf and plant [N] in cereals, it is well known that the biomass increase can stimulate N uptake (Tausz *et al*., 2017; Tausz-Posch *et al*., 2020). This could be the case in our study at this growth stage as NUR was increased by 18% (not significantly) by CETE compared with CATA plants (Fig. 5A), together with the root biomass gain (Yoldi-Achalandabaso *et al*., 2024).

**Table 1.** Effects of development stage, CO_2_ concentration, temperature, water regimen, and consequent treatments on the response of nitrogen metabolism in barley

GS	CO_2_	Temp	EC	WR	Treat	[Leaf N]	[Leaf TSP]	[Leaf FAA]	[Root N]	[RootTSP]	[Root FAA]	[Plant N]
**VEG**	**400**	**23**/**17**	**CATA**	**VC**	**CATA-VC**	52.74±0.61 a	90.21±3.25b	0.25±0.01 b	24.29±2.70 a	21.36±1.46 a	0.07±0.00 bc	43.37±1.13 a
				**VD**	**CATA-VD**	35.63±0.96 c	79.90±2.23c	0.18±0.01 c	18.87±0.62 ab	19.83±0.48 abc	0.06±0.01 bcd	29.25±0.87 d
	**400**	**26**/**20**	**CATE**	**VC**	**CATE-VC**	51.90±0.52 a	105.34±1.10a	0.25±0.01 b	18.84±1.06 ab	19.42±0.67 abc	0.09±0.00 b	41.64±0.42 a
				**VD**	**CATE-VD**	44.42±2.22 b	91.95±1.50b	0.13±0.02 cd	19.71±0.25 ab	15.58±0.33 d	0.13±0.02 a	34.26±1.31 bc
	**700**	**23**/**17**	**CETA**	**VC**	**CETA-VC**	45.03±2.25 b	75.16±1.98 c	0.25±0.01 b	17.43±1.10 b	16.88±0.3 1cd	0.03±0.00 d	35.74±1.82 b
				**VD**	**CETA-VD**	34.53±1.07 c	62.14±2.44 d	0.11±0.01 d	17.05±0.89 b	16.35±0.34 d	0.03±0.00 d	28.83±0.57 d
	**700**	**26**/**20**	**CETE**	**VC**	**CETE-VC**	52.67±0.91 a	90.92±1.03b	0.33±0.01 a	18.77±0.92 ab	20.80±0.24 ab	0.05±0.01 bcd	42.50±0.87 a
				**VD**	**CETE-VD**	40.08±0.22 bc	72.59±2.32c	0.29±0.02 ab	16.99±0.11 b	18.09±0.17 bcd	0.05±0.00 cd	30.92±0.42 cd
**ANTH**	**400**	**23**/**17**	**CATA**	**AC**	**CATA-AC**	41.26±1.10 abc	83.52±5.23a	0.16±0.00 a	18.56±0.05 abc	13.07±0.75 ab	0.06±0.01 cde	35.84±0.78 a
				**AD**	**CATA-AD**	38.28±1.48 abc	49.08±5.32 c	0.15±0.00 a	20.48±0.38 ab	14.48±0.96 a	0.12±0.01 ab	33.35±0.81 a
	**400**	**26**/**20**	**CATE**	**AC**	**CATE-AC**	45.41±0.10 a	64.10±4.07 bc	0.09±0.00 b	17.51±1.09 bc	8.54±0.28 d	0.07±0.02 bcde	34.44±1.29 a
				**AD**	**CATE-AD**	42.81±0.57 ab	52.03±3.54 c	0.06±0.00 d	22.40±1.88 a	11.18±0.37 bcd	0.08±0.01 bcd	34.57±0.77 a
	**700**	**23**/**17**	**CETA**	**AC**	**CETA-AC**	39.84±1.35 abc	83.14±2.67 a	0.06±0.00 d	18.56±0.40 abc	9.87±0.69 cd	0.02±0.00 e	31.63±1.34 ab
				**AD**	**CETA-AD**	37.73±1.66 bc	70.49±2.47 ab	0.07±0.00 cd	20.78±0.70 ab	11.02±0.47 bcd	0.04±0.00 de	31.59±0.64 ab
	**700**	**26**/**20**	**CETE**	**AC**	**CETE-AC**	34.01±3.47 cd	73.42±3.2 0ab	0.08±0.01 bcd	15.55±1.21 c	12.76±0.69 abc	0.10±0.01 abc	25.78±1.75 c
				**AD**	**CETE-AD**	27.61±0.53 d	47.89±3.85 c	0.09±0.01 bc	18.67±1.03 abc	14.25±0.50 a	0.14±0.01 a	26.90±0.99 bc

Barley plants were subjected to different environmental conditions (CATA, current CO_2_ and temperature; CATE, current CO_2_ and elevated temperature; CETA, elevated CO_2_ and current temperature; CETE, elevated CO_2_ and temperature) and water regimes (VC, vegetative control; VD, vegetative drought; AC, anthesis control; AD, anthesis drought). Values represent the mean ±SE of four biological replicates. Different letters indicate significant differences (*P*≤0.05) between water regime treatments and the environmental conditions. Values were analysed separately for the vegetative (VEG) and the anthesis (ANTH) stage. GS, development stage; CO_2_, CO_2_ concentration; WR, water regime; Treat, treatments; [Leaf N], leaf N concentration; Leaf TSP, leaf total soluble protein concentration; [Leaf FAA], free amino acid concentration; [Root N], root N concentration; [Root TSP], root total soluble protein concentration; [Root FAA], root free amino acid concentration; and [Plant N], plant N concentration.

Units for parameters are: [Leaf N], [Root N], and [Plant N], mg N g^–1^ organ; leaf and root TSP, mg Prot g^−1^ DW; leaf and root FAA, mmol Gln g^−1^ DW.

**Table 2.** Effects of development stage, CO_2_ concentration, temperature, water regimen, and consequent treatments on leaf net CO_2_ assimilation rate, night respiration rate, HPR, and GO activities, and leaf senescent level

GS	CO_2_	Temp	EC	WR	Treat	*A*_net_	*R*_n_	HPR	GO	Senescent %
**VEG**	**400**	**23**/**17**	**CATA**	**VC**	**CATA-VC**	612.19±14.05 b	27.72±1.20 c	5.11±0.32 cd	5.83±0.17 bc	—
				**VD**	**CATA-VD**	471.63±8.97 d	36.27±0.96 a	8.04±0.576 b	6.42±0.24 ab	—
	**400**	**26**/**20**	**CATE**	**VC**	**CATE-VC**	542.17±12.32 c	21.18±2.19 d	10.71±0.43 a	6.49±0.39 ab	—
				**VD**	**CATE-VD**	452.25±14.86 d	33.12±1.71 b	10.46±0.62 a	7.47±0.27 a	—
	**700**	**23**/**17**	**CETA**	**VC**	**CETA-VC**	599.99±18.09 b	25.16±1.57 c	4.69±0.19 d	4.72±0.2 3 cd	—
				**VD**	**CETA-VD**	536.44±16.12 c	27.68±1.50 c	6.63±0.41 bc	4.21±0.30 d	—
	**700**	**26**/**20**	**CETE**	**VC**	**CETE-VC**	679.83±26.37 a	36.60±1.66 a	5.34±0.33 cd	3.63±0.17 d	—
				**VD**	**CETE-VD**	565.21±21.35 c	22.50±0.20 d	5.54±0.08 cd	4.51±0.15 d	—
**ANTH**	**400**	**23**/**17**	**CATA**	**AC**	**CATA-AC**	590.70±22.44 bc	12.60±0.65 cd	8.78±0.40 bc	4.77±0.30 b	47.07±2.47c
				**AD**	**CATA-AD**	253.38±14.17 e	18.38±1.32 bcd	10.47±0.70ab	4.98±0.43 b	72.87±3.44a
	**400**	**26**/**20**	**CATE**	**AC**	**CATE-AC**	469.78±12.79 d	19.67±1.05 bc	10.34±0.46ab	6.85±0.49 a	48.04±3.66c
				**AD**	**CATE-AD**	315.70±11.84 e	29.63±0.70 a	12.38±0.42a	7.25±0.42 a	54.40±3.27bc
	**700**	**23**/**17**	**CETA**	**AC**	**CETA-AC**	629.42±22.26 ab	11.78±0.93 d	5.48±0.31cd	4.38±0.27 bc	35.19±5.12d
				**AD**	**CETA-AD**	507.77±26.62 cd	16.80±0.96 bcd	6.02±0.36 cd	4.36±0.44 bc	53.76±3.90bc
	**700**	**26**/**20**	**CETE**	**AC**	**CETE-AC**	681.07±10.86 a	23.48±1.05 ab	3.95±0.34 d	3.47±0.21 bc	40.98±3.79cd
				**AD**	**CETE-AD**	452.08±16.83 d	29.78±1.99 a	7.69±0.75 bc	2.82±0.31 c	61.55±5.54b

Barley plants were subjected to different environmental conditions (CATA, current CO_2_ and temperature; CATE, current CO_2_ and elevated temperature; CETA, elevated CO_2_ and current temperature; CETE, elevated CO_2_ and temperature) and water regimes (VC, vegetative control; VD, vegetative drought; AC, anthesis control; AD, anthesis) drought). Values represent the mean ±SE of four biological replicates. Different letters indicate significant differences (*P*≤0.05) between water regime treatments and the environmental conditions. Values were analysed separately for the vegetative (VEG) and the anthesis (ANTH) stage.

GS, development stage; CO_2_, CO_2_ concentration; WR, water regime; Treat, treatments.

Units for parameters are: *A*_net_, µg CO_2_ m^−2^ s^−1^; *R*_n_, µg CO_2_ m^−2^ s^−1^; HPR and GO, µmol NADH oxidized mg protein^−1^ h^−1^.

**Fig. 6.**
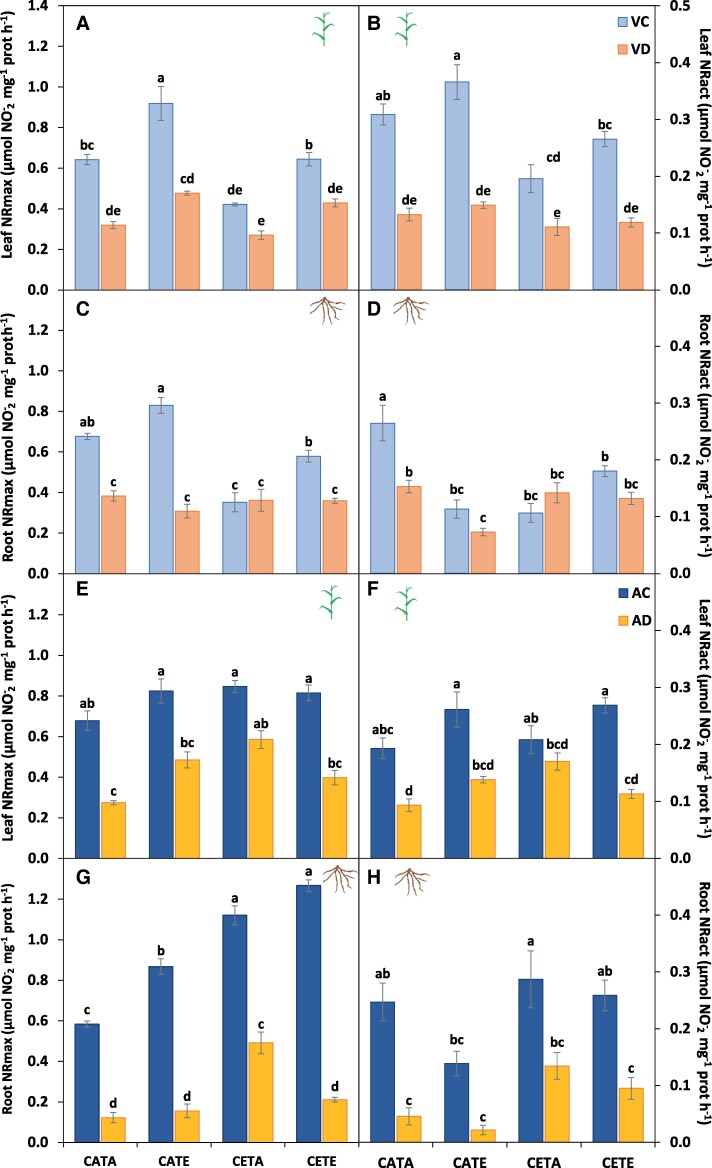
Maximum nitrate reductase (NRmax) and actual nitrate reductase (NRact) activities. (A, B, E, F) Barley leaves and (C, D, G, H) roots at the vegetative (A–D) and anthesis (E–H) stage. The scale for the NRmax and NRact activities is adapted to each maximum value in order to better analyse the results. Barley plants were subjected to different environmental conditions (CATA, current CO_2_ and temperature; CATE, current CO_2_ and elevated temperature; CETA, elevated CO_2_ and current temperature; CETE, elevated CO_2_ and temperature) and water regimes (VC, vegetative control; VD, vegetative drought; AC, anthesis control; AD, anthesis drought). Values represent the mean ±SE of four biological replicates. Different letters indicate significant differences (*P*≤0.05) between water regime treatments and the environmental conditions. Values were analysed separately for the vegetative and anthesis stages and for leaf and root.

**Fig. 7.**
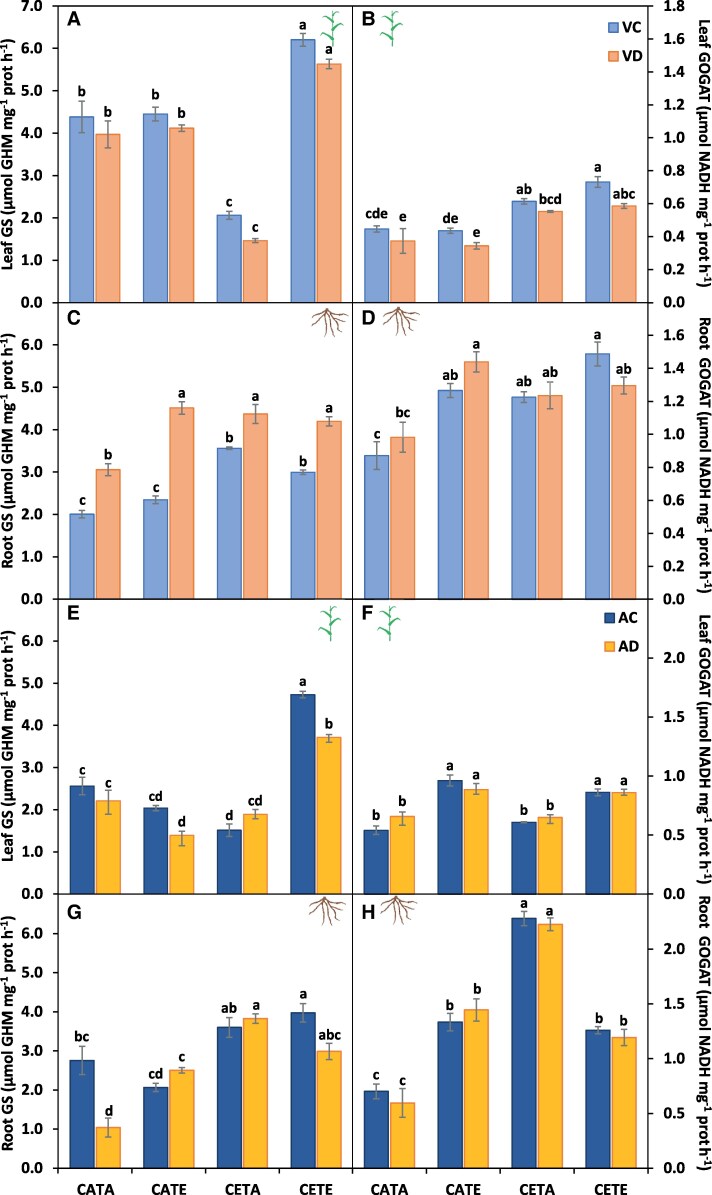
Glutamine synthetase (GS) and glutamate synthase (GOGAT) activities. (A, B, E, F) Barley leaves and (C, D, G, H) roots at the vegetative (A–D) and anthesis (E–H) stage. Barley plants were subjected to different environmental conditions (CATA, current CO_2_ and temperature; CATE, current CO_2_ and elevated temperature; CETA, elevated CO_2_ and current temperature; CETE, elevated CO_2_ and temperature) and water regimes (VC, vegetative control; VD, vegetative drought; AC, anthesis control; AD, anthesis drought). Values represent the mean ±SE of four biological replicates. Different letters indicate significant differences (*P*≤0.05) between water regime treatments and the environmental conditions. Values were analysed separately for the vegetative and anthesis stages and for leaf and root.

Furthermore, the general N reduction and assimilation capacity of CETE plants was boosted compared with CATA under both irrigated and vegetative drought conditions. Indeed, the NR activity was not altered by CETE conditions for leaves (Fig. 6B), even though the root NRact was slightly reduced (Fig. 6D). However, the leaf and root GS/GOGAT activities showed a significant boost (Fig. 7A–D). CETE increased leaf GS and GOGAT activities by 42% and 62% compared with CATA, and the root GS and GOGAT activities by 40%. Robredo *et al*. (2011) obtained similar results for barley leaves at the vegetative stage under the combination of ECO_2_ and drought conditions. In that study, they related the higher leaf GS activity with the boosted NR activity and *A*_net_ rates shown in a companion study (Robredo *et al*., 2010), linking, therefore, the higher sugar levels with the increased N assimilation as Pérez-López *et al*. (2013) demonstrated. This was not exactly the case for CETE, as the NR activity remained unchanged under both irrigated and drought conditions compared with CATA. Nevertheless, the higher *A*_net_ rates of CETE plants (Table 2) could have allowed the production of more C skeletons, reducing power and energy required to increase the N assimilation by the GS/GOGAT cycle and sustain the N requirements to match the biomass gain. This could be consistent with the results of Vicente *et al*. (2018), who observed co-expression between the transcript levels of genes related to N uptake and assimilation and the genes of the respiratory pathway providing energy and C skeletons, through correlation networks and hierarchical clusters on wheat flag leaves. The response of CETE was unique and not comparable with the single stress response (Fig. 2A). However, even if the response of CETE was governed by the effect of ECO_2_ as revealed above, the response of CETA (sole ECO_2_) differed from that of CETE. CETA-VC treatment in the PCA was clustered together with CETA-VD treatment, unlike the rest of the environmental conditions (Fig. 2A). Barley CETA plants did develop a significant reduction in the N status at the vegetative stage (Table 1) in agreement with the extensive evidence for C_3_ species (Wang *et al*., 2012; Feng *et al*., 2015), probably owing to a different non-overlapping cause. Firstly, the N uptake of CETA irrigated and vegetative drought plants was hampered compared with CATA plants. Although a non-significative reduction on NUR was observed (24%; Fig. 5A), the root dry weight was significantly reduced (Yoldi-Achalandabaso *et al*., 2024), which overall would denote a lower N uptake capacity of CETA plants. Secondly, the GVA (Fig. 4A) showed that the leaf biomass of CETA plants increased, while the N uptake decreased, leading to a biomass dilution effect. However, the massive lower N status observed for CETA, mainly for the vegetative drought treatment (Table 1), could not be explained only by the above-mentioned facts. Therefore, we are left with the option of physiological readjustment (Tausz-Posch *et al*., 2020, and references therein).

The N assimilation activity of CETA irrigated and vegetative drought was hampered compared with CATA. The leaf and root NR activities (Fig. 6A–D), together with the leaf GS activity (Fig. 7A), were significantly reduced, although the root GS (Fig. 7C) and GOGAT (Fig. 7D) were increased, probably as a trade-off for the reduced leaf activities, as Andrews *et al*. (2020) and Bloom *et al*. (2020) proposed. However, this enhancement of root ammonium assimilation was not enough to compensate for the decrease in leaf N assimilation. In agreement with Dias de Oliveira *et al*. (2013, 2015b) and Abdelhakim *et al*. (2021), the main explanation for the different response of N metabolism to ECO_2_ between CETE and CETA could be the divergent sink strength capacity. Ainsworth *et al*. (2004) defined the sink strength of a plant ‘as the parts of the plants that, at a given stage of development, are utilizing photosynthates in construction, storage or respiration’. In fact, looking at this issue in more depth, we observed a lower root biomass in CETA than in CETE or even than in CATA (Yoldi-Achalandabaso *et al*., 2024), and lower *R*_n_ rates (Table 2), which could induce a lower sink strength. Consequently, lower *A*_net_ rates were observed in CETA than in CETE plants under well-watered conditions (Table 2). In the case of CETE, the higher *R*_n_ rates could provide the energy necessary for the dark activity of asparagine synthetase (AS) and thus store and/or transport reduced N in the form of asparagine from the source to the most demanding sinks, favouring shoot and root growth. In this respect, and from a theoretical point of view, it is well established that a low ratio between reduced C and high organic N stimulates AS activity (Lam *et al*., 1996). Although we did not measure the asparagine activity or the asparagine concentration, we observed a statistically significant higher concentration of [leaf FAA] on CETE compared with CETA, and a not statistically different greater [root FAA] (Table 1). This fact, together with the above-mentioned higher *R*_n_ rates (Table 2), could serve as a proxy to support the higher AS activity/concentration by CETE, avoiding PAC. The lack of enough sink strength of CETA plants compared with CETE plants resulted in PAC development (lower *V*_Cmax_; Supplementary Table S2). In this regard, Vicente *et al*. (2015) observed similar results for wheat leaves, associating the PAC and reduced N levels with a diminished transcription of the genes involved in photosynthesis and N assimilation. This fact could have triggered the observed reduction in the N assimilation capacity (Fig. 7A, B), contributing, therefore, to the recorded lower N status as Vicente *et al*. (2015) concluded for wheat through down-regulation.

On the other hand, CATE (sole ET environmental condition) did not alter N uptake (Fig. 5A), its reduction (Fig. 6A–D), or its assimilation (Fig. 7A–D) compared with CATA, as Jauregui *et al*. (2015) observed for wheat. Moreover, it is striking that CATE even increased the [leaf TSP] (Table 1). Furthermore, the LNC of CATE was increased compared with CATA, whereas the PNC was reduced (Supplementary Table S1), indicating that the N distribution in CATE changed towards the leaves. This fact could be ascribed to the need for heat-shock protein (HSP) synthesis to protect the Rubisco complex (Hatfield *et al*., 2011). In this regard, it is worth highlighting that a significant reduction in *A*_net_ rates by CATE compared with CATA was observed (Table 2). This decrease in assimilation may be partially due to electron transport inhibition (lower *J*_max_; Supplementary Table S2), which is temperature dependent (von Caemmerer, 2000), but also to a decrease in Rubisco activation (Feller *et al*., 1998). The decrease in the carboxylation efficiency by the ET could be principally related to the thermosensitivity of Rubisco activase, with its activity being reduced (Sage *et al*., 2008). Nevertheless, in the case of CETE, the higher ECO_2_ levels compared with CATE could allow CETE plants to avoid the ET constraints on the *A*_net_ rates (Chavan et al., 2019).

### At the anthesis stage, the N status of barley is reduced under CETE conditions despite the higher N assimilation

As the development of barley continued, the response of N metabolism to drought, ET, and ECO_2_ became more complex. Lower leaf and plant [N] values were recorded at anthesis for CETE compared with CATA, especially under drought conditions (AD; Table 1), despite the lower leaf relative water content and greater decrease in leaf water potential and stomatal conductance observed at CATA (Yoldi-Achalandabaso *et al*., 2024). At this stage, the importance of drought treatment was lower than at the vegetative stage. The PCA clearly highlighted the ECO_2_ as the main factor impacting the N status of CETE plants (Fig. 3A–D). Looking at the GVA (Fig. 4B), and focusing on the AC treatment to better analyse the joint effect of ECO_2_ and ET, the CETE treatments recorded a higher relative leaf biomass than CATA treatments, but lower LNC and, above all, lower [leaf N]. This finding suggests that the decline in the N status of CETE plants cannot be solely attributed to the dilution effect hypothesis caused by the increased biomass. Therefore, to explain our results, other hypotheses must be taken into account. Regarding the mass flow hypothesis, we observed a non-statistically significant 29% lower NUR in CETE compared with CATA which would denote a tendency to decrease N uptake (Fig. 5C). This reduction was highly correlated with the lower stomatal conductance and the consequent lower transpiration that we previously observed (Yoldi-Achalandabaso *et al*., 2024). Moreover, it is worth highlighting that plants take up N by the roots passively via mass flow transpiration, but also actively through specialized transporters (White, 2012). In fact, based on the transcriptomic results of various studies, there is a consensus that ECO_2_ appears to down-regulate the expression of N transporters (Gojon *et al*., 2023, and references therein). In the case of the double action of ECO_2_×ET, Jauregui *et al*. (2015) observed a repression of the expression of NRT1.1 and 1.2 transporters in wheat, attributing the effect to the ECO_2_ which correlated with the diminished N assimilation. These transporters are regulated by the FAA concentration, mainly by glutamine; in fact, when reduced N levels are high, NO_3_^−^ uptake is inhibited. Otherwise, a signal is triggered which induces NO_3_^−^ uptake when the plants sense a lack of it (Miller *et al*., 2008). In our case, we did not directly measure the expression of the above-mentioned transporters. However, the [leaf FAA] was lower in CETE than in CATA, but specifically, no differences in [root FAA] were observed (Table 1). Therefore, based on these results, it seems that the reduction in NO_3_^−^ uptake was not due to the negative regulation of transporters, although we lack enough information to rule this out.

Furthermore, the implication of the lower photorespiration rates for the reduced [N] of CETE plants at anthesis must be borne in mind (Table 2). This process is linked to leaf N metabolism (Bloom, 2015), being, for instance, the main source of production of serine, a key amino acid (Ros *et al*., 2014). Rachmilevitch *et al*. (2004) and Bloom *et al*. (2010) pointed out the NR activity as the limiting factor for the lower N concentration under ECO_2_ due to the lower NADH produced by the malate valve flux from chloroplast to the cytosol, with the reduced photorespiration activity being involved. This hypothesis seems to be unlikely in our case as the NRact activities were not altered (Fig. 6E–H), nor were leaf NO_3_^−^ levels (Supplementary Table S3). In a novel way, Busch *et al*. (2018) gave another interpretation of the photorespiratory pathway considering it is not as closed, as was considered before, and that intermediary molecules could enter other pathways instead of being recycled back to ribulose bisphosphate (RuBP). In addition, in recent years through different models, it also has been suggested that the lower 2-OG produced, triggered by the reduced photorespiration rates, could be involved negatively in N assimilation (Zhao *et al*., 2021). Both Bloom (2015) and Zhao *et al*. (2021) propose that ECO_2_ reduces the NADH/NAD ratio in the cytosol, which would cause the accumulation of nitrate and ammonium, and the reduction in photorespiration decreases the availability of 2-OG necessary for GS/GOGAT activity. However, our results show that the levels of nitrate and ammonium did not increase (Supplementary Table S3), but the rates of GS/GOGAT (Fig. 7E–H) were higher in CETE than in CATA. Thus, from our pieces of evidence, it is difficult to explain the involvement of the lower photorespiration activity in the lower N status. In order to elucidate this, further research is needed to obtain empirical data to support or reject the last hypothesis suggested for the given role of photorespiration in *de novo* assimilation of N.

**Fig. 8.**
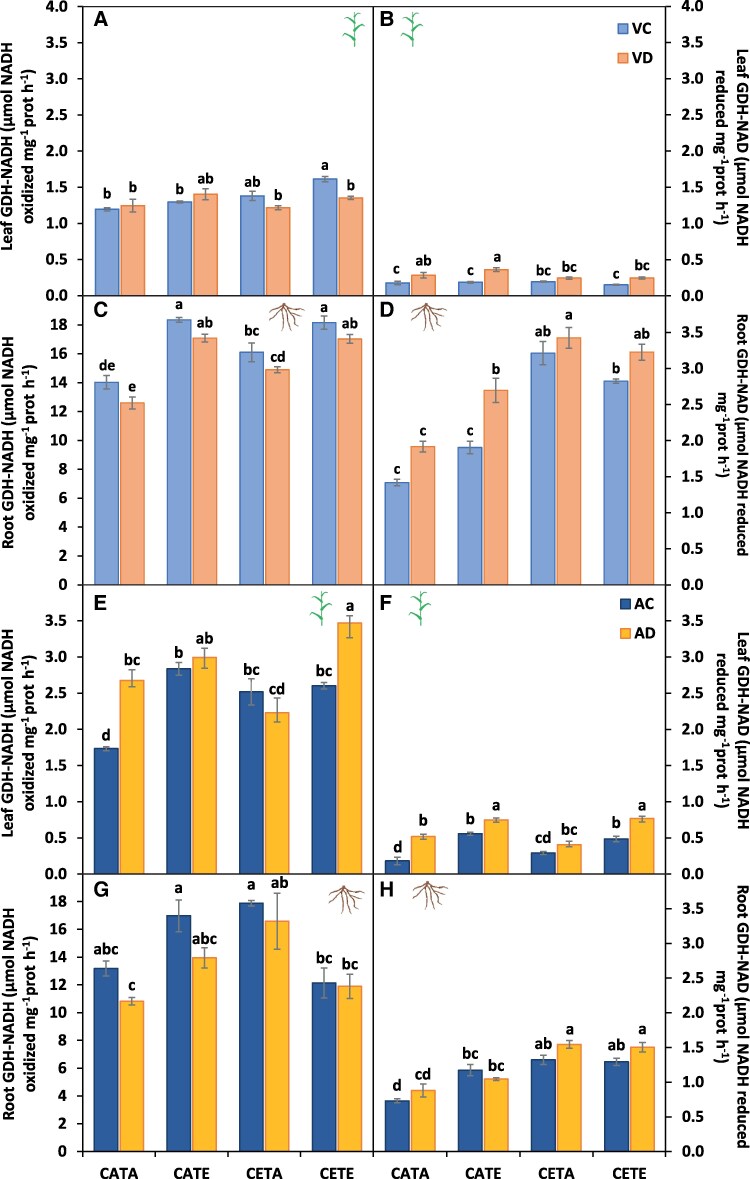
Aminating glutamate dehydrogenase (NADH-GDH) and deaminating glutamate dehydrogenase (NAD-GDH) activities. (A, B, E, F) Barley leaves and (C, D, G, H) roots at the vegetative (A–D) and anthesis (E–H) stage. Barley plants were subjected to different environmental conditions (CATA, current CO_2_ and temperature; CATE, current CO_2_ and elevated temperature; CETA, elevated CO_2_ and current temperature; CETE, elevated CO_2_ and temperature) and water regimes (VC, vegetative control; VD, vegetative drought; AC, anthesis control; AD, anthesis drought). Values represent the mean ±SE of four biological replicates. Different letters indicate significant differences (*P*≤0.05) between water regime treatments and the environmental conditions. Values were analysed separately for the vegetative and anthesis stages and for leaf and root.

Finally, it is important to highlight the recent role attributed to the root in N assimilation under elevated CO_2_ conditions (Andrews *et al*., 2020; Bloom *et al*., 2020). In our study, higher root N assimilation activities under CETE (and CETA) compared with CATA were observed (Fig. 7E–H) in both GS and GOGAT activities and especially in NAD-GDH activity (Fig. 8H). This trend was supported by Adavi and Sathee (2021) under sole ECO_2_ conditions, where they observed an up-regulation in expression of the root GDH gene in a wheat cultivar under high N supply at the seedling stage. This last enzyme is mainly related to the oxidative deamination of glutamate, giving as a product 2-OG and NADH (Vega-Mas *et al*., 2024). Therefore, the increase in this activity could be attributed to an attempt by barley to provide more C skeletons, to fix ammonium, and to use it in respiration as Wang *et al*. (2013) suggested. In this way, the C skeletons and energy and/or reducing power produced could explain the increased root GS and GOGAT activities observed by us (Fig. 3E–H) to try to overcome the decreased plant N status under future drought conditions (Table 1).

Interestingly, as happened for the vegetative stage, the N status of CETE differed from the response of the sole stresses, whereas the N status of CETA was unaffected at this stage, while that of CATE was hampered due to the different *A*_net_ rates (Table 2). As concerns CETA, non-statistically significant differences were observed for the NUR (Fig. 5C), NR activities (Fig. 6E–H), and *A*_net_ rates (Table 2) compared with CATA, whereas the lower leaf GS activity was partially compensated by the higher root GS and GOGAT activities (Fig. 7E–H). The lack of a reduction in the N status by CETA (Fig. 4B), unlike for the vegetative stage, could be due to an internal adjustment of C and N metabolism over time (Zhao *et al*., 2021). However, at this stage (9 d after anthesis), also the source/sink relationship between the vegetative organs and the ear, as well as the senescent rate, must be taken into account (Bogard *et al*., 2011). In fact, the rate at which N remobilization and plant senescence happen is highly correlated with ear demand (Martre *et al*., 2006). In this regard, a significant greater stay-green capacity was observed for CETA leaves compared with CATA and CETE (Table 2), which went hand in hand with the lower ear formation at anthesis by the former (2–3 ears versus 4 ears). In addition, the *V*_Cmax_ was also significantly lower (Supplementary Table S2). Therefore, it cannot be ruled out that the lack of differences in the N status by CETA compared with CATA at anthesis was masked due to its lower senescence and N remobilization rate.

Regarding CATE, the NUR was significantly reduced compared with CATA (Fig. 5C), altering the N reduction and N assimilation processes (Figs 6–8), giving as a result lower [leaf TSP] (Table 1). This fact was not due to a higher senescent level in CATE (Table 2), or to lower leaf GS levels (Fig. 7E), but it was triggered by the higher constraints in the *A*_net_ rates over time (Table 2), probably due to the increased thermosensitivity of Rubisco activase (Sage *et al*., 2008). In agreement with our results, other authors have demonstrated lower *A*_net_ rates under ET over time for wheat (Sharma *et al*., 2015; Abdelhakim *et al*., 2021). In addition, an increase in *R*_n_ was shown (Table 2), as in the NAD-GDH activity (Fig. 8F). The increase in these parameters could be an attempt by CATE plants to obtain energy also for use in defensive and antioxidant processes such as the synthesis of HSPs, providing evidence that the effect of the ET on plants was greater as the plant cycle progressed, with the reproductive stage being the most sensitive developmental stage (Slattery and Ort, 2019). In the case of CETE, the avoidance of Rubisco activity down-regulation caused by ET could be ascribed to the possible effect that ECO_2_ had on the thermal displacement of the *A*_net_ optimum, a concept defined by Long (1991). This fact could be explained by the kinetic properties of Rubisco and the better employment of photoassimilates in development of the defensive system. Plants would have acquired thermotolerance (Wang *et al*., 2008) which protected the enzyme kinetics from the constraints of ET (Gutiérrez *et al*., 2009), increasing RuBP regeneration and avoiding the down-regulation of Rubisco activity (Chavan et al., 2019).

### At maturity, the grain quality will be constrained under CETE conditions due to the negative effect of ET on grain formation

**Fig. 9.**
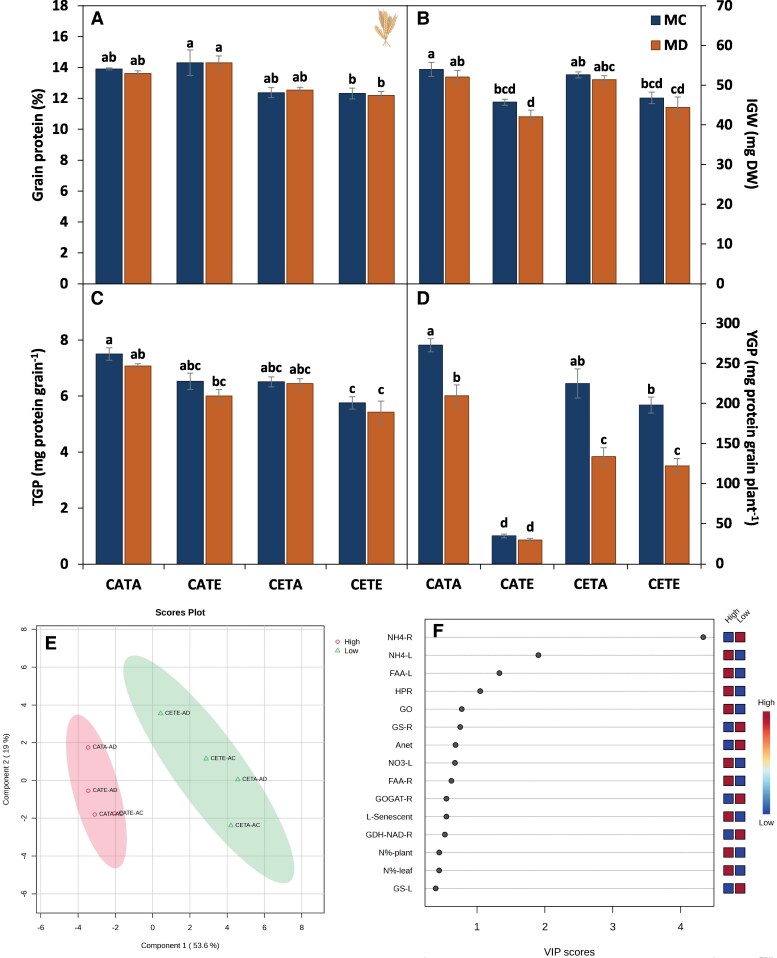
Grain characteristics at the maturity stage. Grain protein concentration (grain protein %; A), individual grain weight (IGW; B), grain protein by grain (TGP; C), and plant yield grain protein (YGP; D). A PLS-DA model (E) and VIP scores (F) that explain which of the studied traits regarding N and C metabolism had more influence on final grain protein concentration are depicted. Based on the results obtained in Fig. 5A, a clustering between high (CATA, CATE) and low (CETE, CETA) grain protein concentrations was carried out. The ellipses of the PLS-DA model represent the 95% confidence interval. The key for the VIP scores is depicted in the figure. Barley plants were subjected to different environmental conditions (CATA, current CO_2_ and temperature; CATE, current CO_2_ and elevated temperature; CETA, elevated CO_2_ and current temperature; and CETE, elevated CO_2_ and temperature) and water regimes (MC, maturity control; MD, maturity drought). Values represent the mean ±SE of four biological replicates. Different letters for (A–D) indicate significant differences (*P*≤0.05) between water regime treatments and the environmental conditions.

With the idea to look at N metabolism in more depth and to go beyond a mere description of the effects of environmental agents, a PLS-DA model was run with the studied parameters at anthesis (Fig. 9E) in order to explain the obtained results for the [grain protein] at maturity. PLS-DA is effective for understanding the variables that differentiate treatments, which can be assessed using methods such as VIP scores (Labory *et al*., 2024). The PCA associated with the PLS-DA (Supplementary Fig. S1) clustered the grain protein concentration in two groups of the eight different treatments (four environmental conditions×two water regimens), which were split by the growth [CO_2_] irrespective of the water treatment. The high [grain protein] was associated with the ACO_2_ conditions regardless of the temperature (CATA and CATE), while the low [grain protein] was linked to ECO_2_ (CETE and CETA). Three main processes were highlighted by the VIP score of the PLS-DA model explaining the differences between the two clusters (Fig. 9F), in agreement with the above-mentioned main hypothesis to explain why ECO_2_ reduces plant or organ N concentration: photoassimilate production; N uptake; and physiological readjustment (Tausz-Posch *et al*., 2020). Specifically, CETE [grain protein] followed the same trend as for leaf and plant N concentration at anthesis, namely a reduction by 15% compared with CATA conditions (Fig. 9A). The close relationship between both growth stages was not surprising since the grain N in cereal crops, such as wheat and barley, comes principally from the stored N in the vegetative organs and subsequent remobilization around anthesis (Kong *et al*., 2016, and references therein). This fact emphasizes the relevance of analysing N metabolism of cereals at the reproductive stage to understand the whole dynamics of N metabolism and not only at the vegetative stage, as Yoldi-Achalandabaso *et al*. (2025) concluded in their review and meta-analysis for crop physiology and agronomical components under triple interaction.

Lower [grain protein] due to ECO_2_ can trigger negative impacts in countries where the main protein source is dependent on grain protein (Myers *et al*., 2014). For bread baking, lower protein levels negatively influence the quality of the product, as stated by Högy and Fangmeier (2008) and Panozzo *et al*. (2014). However, depending on the end use of the grain, the implications could vary. As regards malting barley—the species and cultivar of concern in this study—the reduction in [grain protein] observed can be beneficial as the brewery industry seeks grains with lower protein level (Erbs *et al*., 2010). The [grain protein] under drought conditions was not altered compared with each control (Fig. 9A). Nevertheless, for any end-use of the grain, it is not only the [grain protein] that matters as a quality marker, but also the grain size and grain number (Barnabás *et al*., 2008). In this regard, the negative effect that drought had on grain formation and final grain quality cannot be ruled out (Fig. 9D) compared with the plant N status for the vegetative organs (Table 1). As shown in Table 2, the reduced *A*_net_ rates resulting from drought treatments may potentially hinder the availability of sugars and starch necessary for development of florets, thereby inducing spikelet sterility. As illustrated in Fig. 4D, the decline in total grain biomass, rather than a decrease in grain protein, is the primary factor contributing to the substantial decrease observed in YGP (Fig. 9D).

In addition, under CETE conditions, a significant reduction in IGW was shown compared with CATA, which was due to the negative effects that ET had on grain formation, evidenced by CATE treatment (Fig. 9B). As a result, the reduction in the TGP for CETE conditions was magnified (Figs 4C, 9B). These results are not surprising since, on one hand, barley is not well adapted to ET effects (Mahalingam and Bregitzer, 2019), as both pollen development and fertilization, and grain formation, are hypersensitive to ET (Abiko *et al*., 2005). On the other hand, the impact of ET on final reproductive fitness is closely related to the developmental stage at which it occurs (Gray and Brady, 2016), which in our case encompassed the whole life cycle of the plants. Indeed, Jacott and Boden (2020) concluded for wheat and barley that the maximum temperature for floral transition is 20–25 °C and for spike formation is 20 °C, and in our experiment barley CATE and CETE plants grew at 26/20 °C (day/night). In the case of CETE, the negative effect of the ET on grain formation was partially ameliorated compared with CATE (Fig. 9B), especially in drought conditions. This trend could be ascribed to the positive effect of ECO_2_ on the increase in photosynthetic rates (Table 2) and/or on structural damage amelioration in the grain set (Fig. 9B) as Dias de Oliveira *et al*. (2015a) observed for wheat. Specifically, higher sucrose and hexose availability derived from the higher photosynthetic rates at CETE compared with CATE has been related to a better osmotic adjustment and, therefore, ET tolerance (Wahid *et al*., 2007). However, in CETE, the 30% reduction in the YGP was higher than that of [grain protein] (Fig. 9D), therefore it reflected an alteration in the grain set and yield (Fig. 4D). This outcome agrees with results by Ingvordsen *et al*. (2016) for a panel of >100 barley accessions for ET×ECO_2_ under irrigated conditions and in pot experiments too, which calls for concern. In spite of the scarcity of facilities to carry out field trials under ECO_2_ due to the high costs of the system (Yoldi-Achalandabaso *et al*., 2025), we urge others to replicate this type of experiments under field conditions in order to anticipate the effects of climate change on cereal production, as part of human food security is largely dependent on cereal grain protein (Myers *et al*., 2014).

## Conclusion

**Fig. 10.**
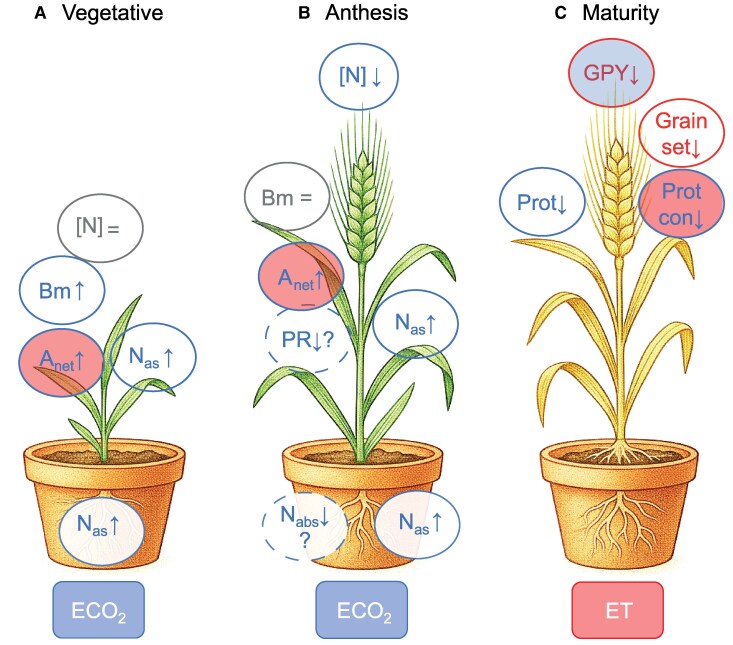
Outline of the main differences in the N status of barley resulting from the joint action of elevated CO_2_ (ECO_2_) and temperature (ET) under drought conditions (CETE versus CATA) at the vegetative, anthesis, and maturity stages. The boxes at the bottom of the figure represent the environmental factor (ECO_2_ or ET) that had the greatest impact on the N status of barley at each developmental stage. The circles represent the key variables that define this N status, and the arrows indicate the direction of the effect. The colour of the letters inside the circles and the border lines indicate which was the primary environmental factor influencing it, blue for CO_2_, red for temperature, and grey for no effect. The colour of filled circles indicates the influence of the secondary environmental factor: blue for CO_2_ and red for temperature. Dotted lines suggest an effect on a certain variable to be investigqated in greater depth in future research. (A) At the vegetative stage, the joint action of ECO_2_ and temperature stimulated the net photosynthetic rate (*A*_net_), driven by the ECO_2_, supplying more C skeletons for energy and reducing power production that barley employed to increase N assimilation (Nas). At this stage, plants were able to balance the N status ([N]) with the biomass increase (Bm). (B) At the anthesis stage, however, barley plants were not able to balance the N status with the higher *A*_net_, although the Nas had increased, mainly in roots. The lower N absorption (Nabs) and photorespiration activity (PR) are suggested as putative target processes to investigate in future research, beyond the dilution effect. (C) Lastly, at maturity, the lower protein concentration (Prot) triggered by the ECO_2_ and the reduced grain protein content (Prot con) and grain set formation mainly caused by the ET gave as a result a reduced grain protein yield (GPY). AI Gemini 2.5 Flash Image was used to create the pots and the plants.

This study provides a novel integrated perspective on plant N metabolism, studying the triple interaction of drought, ECO_2_, and ET. The primary conclusion is that the response of future drought×ECO_2_×ET combined conditions was unique and not comparable with the single or bifactorial stress response. The N metabolism of barley was governed by the fertilizer effect of CO_2_ on C metabolism, while ET constrained grain formation and quality. Furthermore, the response was found to be growth stage-specific (Fig. 10). At the vegetative stage, the N status was not altered, whereas it resulted in reduced levels of N in the plant and the grain at the stages of anthesis and maturity. Despite the augmented N assimilation, attributable to the increased C skeletons, energy, and reducing power levels derived from the higher photosynthetic rates, the N status of barley did not match the biomass gain, due in part to the diminished N uptake, caused by stomatal closure. Nevertheless, the putative negative impact that the lower photorespiration activity could have on the failure to meet the N requirements cannot be ruled out. This fact necessitates an urgent understanding of N and C metabolism in cereals under future drought environmental conditions, in which both CO_2_ and temperature will increase concomitantly.

## Supplementary Material

eraf531_Supplementary_Data

## Data Availability

The primary data supporting this study were not made publicly available at the time of publication. The data that support the findings of this study are available from the corresponding authors upon request.

## Supplementary data

The following supplementary data are available at *JXB* online.

Table S1. Effects of development stage, environmental condition, and water regimen on leaf N content, root N content, and plant N content.

Table S2. Effects of development stage, environmental condition, and water regimen on the maximum rate of carboxylation and the maximum rate of electron transport.

Table S3. Effects of development stage, environmental condition, and water regimen on leaf nitrate, leaf ammonium, root nitrate, and root ammonium concentration.

Fig. S1. Principal component analysis associated with the PLS-DA, where the effect of the studied main factors on N metabolism at the anthesis stage is depicted, clustered by high protein and low protein concentrations.
