# Supplementary material for: The grain protein yield of barley under future drought is modified by the joint action of elevated CO2 and temperature
Source: J Exp Bot. 2025 Dec 2;77(6):1807–26. doi: 10.1093/jxb/eraf531 (PMC13016997; doi:10.1093/jxb/eraf531)
Supplement: eraf531_Supplementary_Data [file eraf531_supplementary_data.pdf]

Supplementary data

Table S1. Effects of development stage (*GS*), environmental condition (*EC*) and water regimen (*WR*) on leaf N content (LNC), root N content (RNC) plant N content (PNC). Barley plants were subjected to different environmental conditions (CATA, current CO_2 and temperature; CATE, current CO_2 and elevated temperature; CETA, elevated CO_2 and current temperature CETE, elevated CO_2 and temperature) and water regimes (VC, vegetative control; VD, vegetative drought; AC, anthesis control; AD, anthesis drought). Values represent the mean ± SE of four biological replicates. Different letters indicate significant differences ($P \leq 0.05$) between water regime treatments and the environmental conditions. Values were analysed separately for the vegetative and the anthesis stage.

GS	CO2	Temp	EC	WR	Treat	LNC	RNC	PNC
**VEG**	**400**	**23/17**	**CATA**	**VC**	**CATA-VC**	18.83 ± 0.70b	5.84 ± 0.59a	36.98 ± 2.07ab
				**VD**	**CATA-VD**	10.14 ± 0.59d	5.74 ± 0.22ab	26.02 ± 3.54cd
	**400**	**26/20**	**CATE**	**VC**	**CATE-VC**	20.36 ± 0.44b	3.72 ± 0.11c	32.58 ± 0.72bc
				**VD**	**CATE-VD**	14.58 ± 1.19c	4.96 ± 0.27abc	26.10 ± 1.21cd
	**700**	**23/17**	**CETA**	**VC**	**CETA-VC**	17.34 ± 1.64bc	4.25 ± 0.36c	30.58 ± 2.34bc
				**VD**	**CETA-VD**	10.14 ± 0.44d	4.53 ± 0.29bc	19.68 ± 0.55d
	**700**	**26/20**	**CETE**	**VC**	**CETE-VC**	26.36 ± 0.42a	5.10 ± 0.21abc	45.22 ± 0.94a
				**VD**	**CETE-VD**	14.42 ± 0.39c	5.04 ± 0.16abc	27.48 ± 0.51cd
**ANTH**	**400**	**23/17**	**CATA**	**AC**	**CATA-AC**	105.02 ± 3.19a	7.69 ± 0.31cd	270.72 ± 9.38a
				**AD**	**CATA-AD**	91.29 ± 3.98ab	10.63 ± 0.69ab	238.99 ± 2.94ab
	**400**	**26/20**	**CATE**	**AC**	**CATE-AC**	100.88 ± 2.40a	5.17 ± 0.62d	189.39 ± 5.30c
				**AD**	**CATE-AD**	94.39 ± 1.90a	8.07± 0.50bc	198.33 ± 7.32c
	**700**	**23/17**	**CETA**	**AC**	**CETA-AC**	93.94 ± 3.81a	7.62 ± 0.34cd	219.15 ± 9.28bc
				**AD**	**CETA-AD**	86.79 ± 4.78ab	8.18 ± 0.77abc	204.66± 5.66bc
	**700**	**26/20**	**CETE**	**AC**	**CETE-AC**	98.66 ± 6.10a	7.94 ± 0.80bc	217.15 ± 11.66bc
				**AD**	**CETE-AD**	72.69 ± 4.55b	10.95 ± 0.78a	195.17 ± 9.39c

Units for next parameters are: LNC, RNC and PNC, mg N $organ^{-1}$.

Table S2. Effects of development stage (*GS*), environmental condition (*EC*) and water regimen (*WR*) on the maximum rate of carboxylation (V_{Cmax}) and the maximum rate of electron transport (J_{max}). Barley plants were subjected to different environmental conditions (CATA, current CO_2 and temperature; CATE, current CO_2 and elevated temperature; CETA, elevated CO_2 and current temperature CETE, elevated CO_2 and temperature) and water regimes (VC, vegetative control; VD, vegetative drought; AC, anthesis control; AD, anthesis drought). Values represent the mean ± SE of four biological replicates. Different letters indicate significant differences ($P \leq 0.05$) between water regime treatments and the environmental conditions. Values were analysed separately for the vegetative and the anthesis stage.

GS	*EC*	*WR*	V_{Cmax}	J_{max}
**VEG**	**CATA**	**VC**	5.71 ± 0.34a	10.47 ± 0.11a
		**VD**	5.53 ± 0.56a	11.03 ± 0.41a
	**CATE**	**VC**	4.94 ± 0.38b	8.89 ± 0.11c
		**VD**	5.13 ± 0.14ab	8.87 ± 0.55bc
	**CETA**	**VC**	4.10 ± 0.46c	9.35 ± 0.28b
		**VD**	3.78 ± 0.37c	8.23 ± 0.33d
	**CETE**	**VC**	5.25 ± 0.34ab	10.74 ± 0.38a
		**VD**	4.59 ± 0.10b	9.03 ± 0.16bc
**ANTH**	**CATA**	**AC**	5.33 ± 0.26b	10.18 ± 0.59b
		**AD**	4.68 ± 0.63bc	9.07 ± 0.35bc
	**CATE**	**AC**	4.45 ± 0.45bc	7.90 ± 0.26d
		**AD**	5.35 ± 0.56ab	8.07 ± 0.55cd
	**CETA**	**AC**	4.11 ± 0.21c	8.84 ± 0.40c
		**AD**	4.36 ± 0.74bc	8.54 ± 0.21
	**CETE**	**AC**	6.14± 0.40a	11.50 ± 0.20a
		**AD**	4.06 ± 0.24c	8.64 ± 0.32c

Units for next parameters are: V_{Cmax}, nmols CO_2 cm^{-2} s^{-1}; J_{max}, nmols e^- cm^{-2} s^{-1}.

Table S3. Effects of development stage (*GS*), environmental condition (*EC*) and water regimen (*WR*) on leaf nitrate (L-NO_3^-), leaf ammonium (L-NH_4^+), root nitrate (R-NO_3^-) and root ammonium (R-NH_4^+) concentration. Barley plants were subjected to different environmental conditions (CATA, current CO_2 and temperature; CATE, current CO_2 and elevated temperature; CETA, elevated CO_2 and current temperature CETE, elevated CO_2 and temperature) and water regimes (VC, vegetative control; VD, vegetative drought; AC, anthesis control; AD, anthesis drought). Values represent the mean ± SE of four biological replicates. Different letters indicate significant differences ($P \leq 0.05$) between water regime treatments and the environmental conditions. Values were analysed separately for the vegetative and the anthesis stage.

GS	*EC*	*WR*	L- NO_3^-	L-NH_4^+	R-NO_3^-	R-NH_4^+
**VEG**	**CATA**	**VC**	396.39 ± 5.61b	559.11 ± 19.47de	79.41 ±1.80c	463.90 ± 22.12bc
		**VD**	219.47 ± 9.08de	704.20 ± 17.05bc	53.27 ± 1.96a	284.44 ± 19.65d
	**CATE**	**VC**	576.00 ± 40.77a	518.80 ± 18.22e	89.10 ± 9.32c	203.08 ± 9.87d
		**VD**	327.52 ± 22.26bc	347.74 ± 23.22f	127.19 ± 3.27bc	187.74 ± 19.00d
	**CETA**	**VC**	234.23 ± 3.15de	690.73 ± 23.00cd	40.89 ± 9.39d	698.95 ± 27.83a
		**VD**	174.75 ± 16.94e	937.75 ± 38.74a	51.98 ± 4.84bc	517.25 ± 68.88b
	**CETE**	**VC**	302.09 ± 14.19cd	846.70 ± 22.37ab	85.38 ± 3.70cd	305.08 ± 24.92d
		**VD**	220.16 ± 11.00de	642.58 ± 59.05cd	60.82 ± 2.68b	313.91± 28.89cd
**ANTH**	**CATA**	**AC**	197.43 ± 5.79a	1128.29 ± 41.60ab	73.33 ± 3.44b	172.32 ± 13.86d
		**AD**	145.53 ± 13.53cd	1074.79± 56.55bc	59.00± 6.01b	184.64 ± 14.55d
	**CATE**	**AC**	197.59 ± 2.94a	918.71± 25.79cd	70.61 ± 6.44ab	176.65 ± 5.53d
		**AD**	189.12 ± 12.04ab	1217.38± 54.60a	113.30 ± 16.15a	184.64 ± 14.55d
	**CETA**	**AC**	184.16 ± 6.90abc	769.81 ± 27.56d	69.98 ± 2.67b	766.03 ± 60.58b
		**AD**	150.74 ± 8.73bcd	940.95 ± 8.43bcd	74.07 ± 7.71b	1061.65 ± 76.56a
	**CETE**	**AC**	179.06± 8.10abcd	809.72 ± 24.91d	79.01 ± 8.05b	627.21± 16.11bc
		**AD**	140.41 ± 2.96d	1013.85 ± 65.57bc	65.13 ± 4.28b	458.57 ± 31.20c

Units for next parameters are: Leaf and Root NO_3^-, µmol NO_3^- gDW^{-1}; Leaf and Root NH_4^+, nmol NH_4^+ gDW^{-1}.

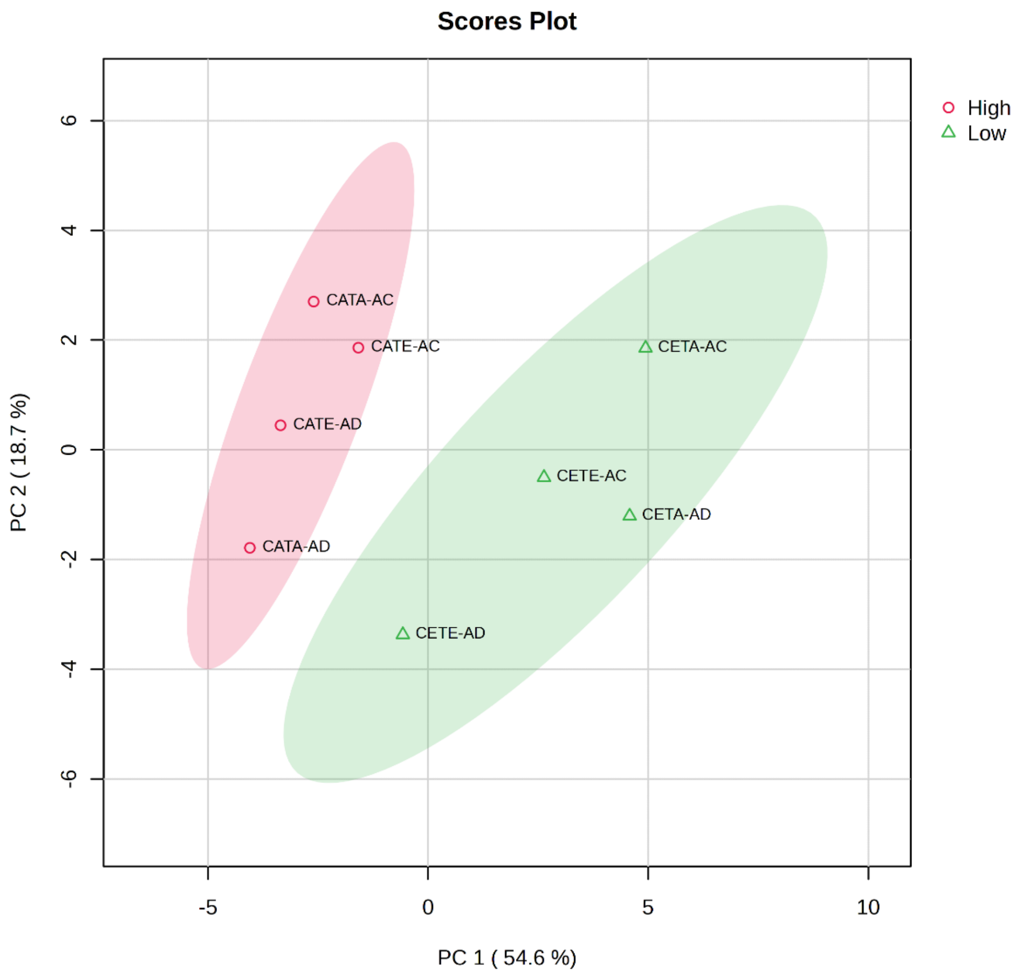

Trait	***p value***
**HPR**	0.0028
**R-NH_4^+**	0.0108
**R-NAD-GDH**	0.0145
**GO**	0.0244
**Plant [N]**	0.0343
**R-GS**	0.0379

Fig. S1. Principal Component Analysis (PCA) for the analysed traits in the MS where the effect of the studied main factors on the nitrogen metabolism at the anthesis stage is depicted, clustered by high protein (CATA, CATE) and low protein (CETE, CETA) concentrations. Below, traits that were statistically significant ($p<0.05$) in the pls-da model.
